# Therapeutic trials for long COVID-19: A call to action from the interventions taskforce of the RECOVER initiative

**DOI:** 10.3389/fimmu.2023.1129459

**Published:** 2023-03-09

**Authors:** Hector Bonilla, Michael J. Peluso, Kathleen Rodgers, Judith A. Aberg, Thomas F. Patterson, Robert Tamburro, Lawrence Baizer, Jason D. Goldman, Nadine Rouphael, Amelia Deitchman, Jeffrey Fine, Paul Fontelo, Arthur Y. Kim, Gwendolyn Shaw, Jeran Stratford, Patricia Ceger, Maged M. Costantine, Liza Fisher, Lisa O’Brien, Christine Maughan, John G. Quigley, Vilma Gabbay, Sindhu Mohandas, David Williams, Grace A. McComsey

**Affiliations:** ^1^ Department of Medicine and Infectious Diseases, Stanford University, Palo Alto, CA, United States; ^2^ Department of Medicine and Infectious Diseases, University of California, San Francisco, San Francisco, CA, United States; ^3^ Center for Innovations in Brain Science, University of Arizona, Tucson, AZ, United States; ^4^ Department of Medicine, Infectious Diseases, Icahn School of Medicine at Mount Sinai, Chief, Division of Infectious Disease, New York, NY, United States; ^5^ Department of Medicine, Infectious Diseases, The University of Texas Health Science Center at San Antonio, San Antonio, TX, United States; ^6^ Division of Intramural Research, National Institute of Health, Bethesda, MD, United States; ^7^ National Heart Lung and Blood Institute, Division of Lung Diseases, National Institutes of Health, Bethesda, MD, United States; ^8^ Department of Medicine, Organ Transplant and Liver Center, Swedish Medical Center, Seattle, WA, United States; ^9^ Division of Allergy and Infectious Diseases, University of Washington, Seattle, WA, United States; ^10^ Department of Medicine, Division of Infectious Diseases, Emory University School of Medicine, Atlanta, GA, United States; ^11^ Department of Clinical Pharmacy, University of California, San Francisco, San Francisco, CA, United States; ^12^ Department of Rehabilitation Medicine at New York University (NYU) Grossman School of Medicine, Physical Medicine and Rehabilitation Service, New York University (NYU), New York University Medical Center, New York, NY, United States; ^13^ Applied Clinical Informatics Branch, National Library of Medicine, National Institutes of Health, Bethesda, MD, United States; ^14^ Department of Medicine at Harvard Medical School, Division of Infectious Disease, Boston, MA, United States; ^15^ Research Triangle Institute (RTI), International, Durham, NC, United States; ^16^ Department of Obstetrics and Gynecology, The Ohio State University, Columbus, OH, United States; ^17^ Long COVID Families, Houston, TX, United States; ^18^ Utah Covid-19 Long Haulers, Salt Lake City, UT, United States; ^19^ Department of Medicine, University of Illinois at Chicago, Chicago, IL, United States; ^20^ Department of Medicine, Albert Einstein College of Medicine, New York, NY, United States; ^21^ Department of Pediatrics, Keck School of Medicine, University of Southern California, Los Angeles, CA, United States; ^22^ Department of Medicine, University of Michigan, Ann Arbor, MI, United States; ^23^ Department of Pediatrics and Medicine, Case Western Reserve University, Cleveland, OH, United States

**Keywords:** post-acute sequela of SARS-CoV-2 (PASC), long COVID, SARS- CoV-2, long haulers, treatment, clinical trials, recover

## Abstract

Although most individuals recover from acute SARS-CoV-2 infection, a significant number continue to suffer from Post-Acute Sequelae of SARS-CoV-2 (PASC), including the unexplained symptoms that are frequently referred to as long COVID, which could last for weeks, months, or even years after the acute phase of illness. The National Institutes of Health is currently funding large multi-center research programs as part of its Researching COVID to Enhance Recover (RECOVER) initiative to understand why some individuals do not recover fully from COVID-19. Several ongoing pathobiology studies have provided clues to potential mechanisms contributing to this condition. These include persistence of SARS-CoV-2 antigen and/or genetic material, immune dysregulation, reactivation of other latent viral infections, microvascular dysfunction, and gut dysbiosis, among others. Although our understanding of the causes of long COVID remains incomplete, these early pathophysiologic studies suggest biological pathways that could be targeted in therapeutic trials that aim to ameliorate symptoms. Repurposed medicines and novel therapeutics deserve formal testing in clinical trial settings prior to adoption. While we endorse clinical trials, especially those that prioritize inclusion of the diverse populations most affected by COVID-19 and long COVID, we discourage off-label experimentation in uncontrolled and/or unsupervised settings. Here, we review ongoing, planned, and potential future therapeutic interventions for long COVID based on the current understanding of the pathobiological processes underlying this condition. We focus on clinical, pharmacological, and feasibility data, with the goal of informing future interventional research studies.

## Background

While most people fully recover within weeks of SARS-CoV-2 infection, a subset of individuals experience symptoms that persist well beyond the acute period (1). These symptoms can be debilitating, interfering with return to usual activities and impacting quality of life. While efforts to quantify the scale of the problem are ongoing, there is a growing consensus that post-COVID conditions, collectively known as Post-Acute Sequelae of SARS-CoV-2 infection (PASC), including the unexplained symptoms of long COVID, represent a substantial public health concern. However, there is currently no standard of care for long COVID and no agreed upon treatment. In this article, we review the current state of long COVID management with a view toward therapies with promising early data. Selected studies of pharmaceutical interventions are currently listed in ClinicalTrials.gov* is presented in **Table 1**. We focus on the biological mechanisms that have been identified as potential drivers of this condition.

**Table 1:** Selected pharmaceutical interventions currently listed in ClinicalTrials.gov^*^.

Drug	Drug Class	Proposed Mechanism for PASC	NCT Number
Antiviral			
Favipiravir	SARS-CoV-2 RNA-dependent RNA polymerase inhibitor	Viral clearance or reduction in inflammation	NCT04448119
Nirmatrelvir/ritonavir (Paxlovid)	Protease inhibitor	Viral clearance or reduction in inflammation	NCT05595369 NCT05576662
Remdesivir	SARS-CoV-2 RNA-dependent RNA polymerase inhibitor	Viral clearance	NCT04978259
Cardiac Agents			
Ivabradine	hyperpolarization-activated cyclic nucleotide-gated (HCN) channel blocker	Treat postural orthostatic tachycardia syndrome	NCT05481177
Metoprolol succinate	Beta-1 antagonist	Improve cardiac function	NCT05096884
Antiinflammatory agents			
Cannabinoid containing formulations	Cannabinoids	Antiinflammatory	NCT04997395 NCT05467904
Fluvoxamine	selective serotonin reuptake inhibitor (SSRI)	Improve parosmia	NCT05216614
Ibudilast	Phosphodiesterase Inhibitor	Block Inflammatory pathways	NCT05513560
Imatinib	Kinase inhibitor	Antiinflammatory	NCT05220280
Infliximab	TNF-alpha inhibitor	Antiinflammatory	NCT05220280
Low Dose Naltrexone	Opioid antagonist	Antiinflammatory	NCT04604704 NCT05430152
Pentoxifylline	Xanthine derivative Hemorrheologic Agent	Antiinflammatory/immunomodulator Vasodilator	NCT05513560
Vitamin D	Dietary Supplement	Treat post-COVID Vitamin D deficiency	NCT05633472
Respiratory Agents			
LYT-100 (deupirfenidone)	Antifibrotic	Antifibrotic/antiinflammatory	NCT04652518
Montelukast	leukotriene receptor antagonists	Improve respiratory PASC symptoms	NCT04695704
S-1226	bronchodilator	Improve respiratory PASC symptoms	NCT04949386
Other			
AXA1125	Endogenous metabolic modulator	Improve muscle function	NCT05152849
Lithium	Antimanic agent	Improve fatigue and brain fog	NCT05618587
Pimozide	Dopamine receptor antagonist	Treat tinnitus	NCT05507372
RSLV-132	RNase-Fc fusion protein	Lessen fatigue	NCT04944121
Somatropin	Growth hormone	Resolve associated hormone secretion disorder	NCT03554265
Temelimab	Monoclonal Antibody	Improve cognitive functioning	NCT05497089
TNX-102 (cyclobenzaprine)	Muscle relaxant	Pain	NCT05472090
Vortioxetine	selective serotonin reuptake inhibitor (SSRI)	Improve cognitive functioning	NCT05047952

^*^ Search done on 12/02/2022. This list is not all inclusive and excludes non pharmacological interventions (e.g., physical or cognitive therapy, supplements).

## Case definitions, epidemiology, and clinical features

**Figure 1:**
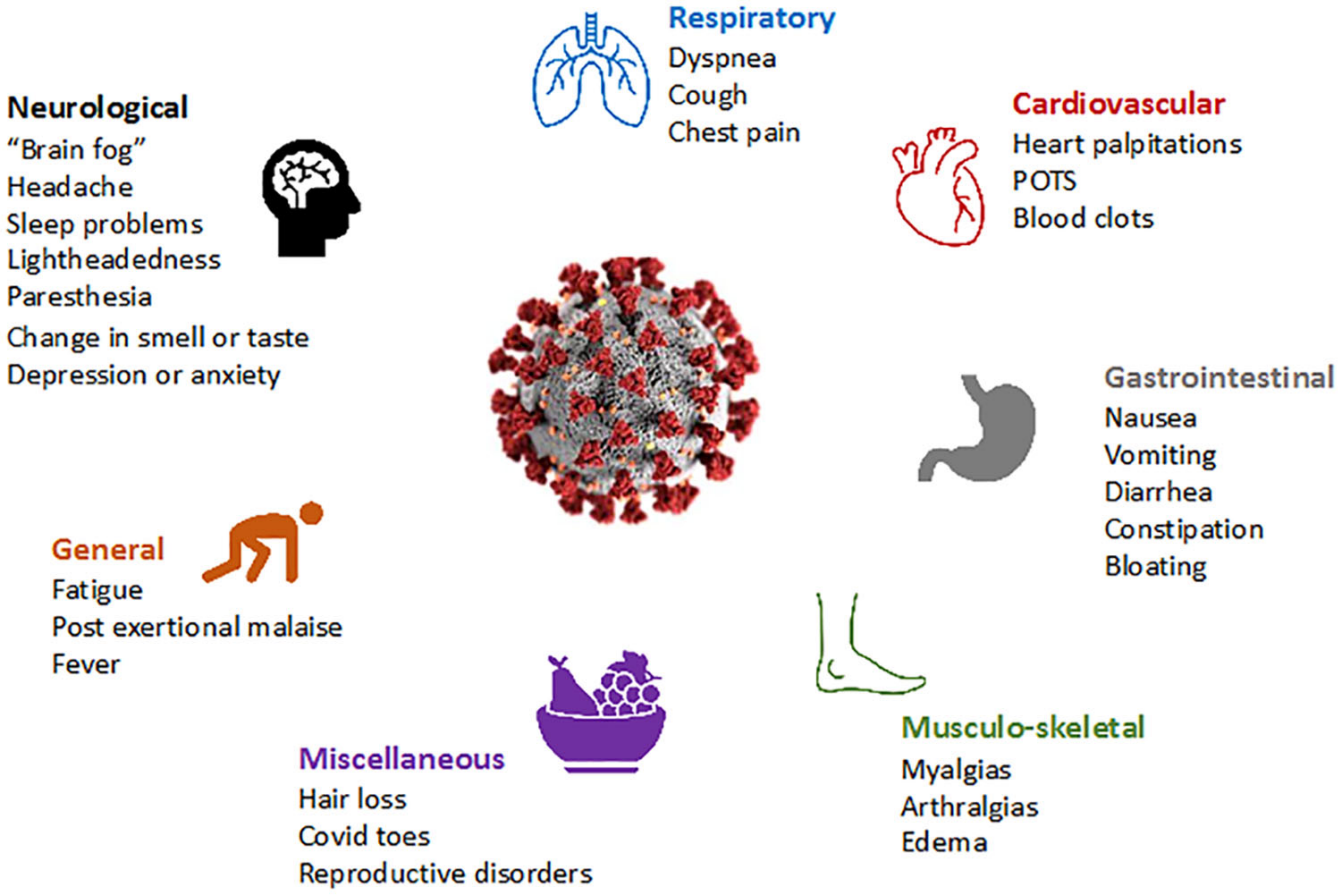
Most common symptoms reported in long COVID population.

Long COVID is increasingly recognized even in people who experience asymptomatic or mild SARS-CoV-2 infection (2). The U.S. Centers for Disease Control and Prevention (CDC) defines long COVID (which they refer to as “post-COVID conditions”) as symptoms persisting more than 28 days after the initial SARS-CoV-2 infection (3), while the U.K. National Institute for Health and Care Excellence (NICE) (4) and the World Health Organization (WHO) require symptoms to have persisted greater than 12 weeks after the initial infection (5). The American Academy of Physical Medicine and Rehabilitation (AAPM&R) estimates that there are more than 29 million people suffering from long COVID in the US as of December 2022 (6). The overall global prevalence of long COVID is estimated at 43% of the acute cases (hospitalized 54% and non-hospitalized 36%) (7). Recent data from the CDC estimate that 7.5% (over 24 million people) of the adult population has long COVID symptoms. In a meta-analysis which found that fatigue/weakness, myalgia/arthralgia, depression, anxiety, memory loss, concentration difficulties, dyspnea, and insomnia, were the most prevalent symptoms (8, 9) (**Figure 1**), and that the prevalence of long COVID is three times higher among 50-59 year-olds than over-80-year-olds (10). Even if the prevalence of debilitating symptoms is low, the overall health burden is large given the scale of the pandemic.

While the clinical features of long COVID and epidemiologic risk factors have been described over the last two years, the underlying biological mechanisms remain elusive. Several hypotheses have been proposed. These include persistence of SARS-CoV-2 reservoirs, presence of microthrombi, induction of autoantibodies, hyperreactive immune activation, reactivation of other latent viral coinfections such as Epstein-Barr Virus (EBV), mitochondrial dysfunction, and gut dysfunction/dysbiosis (11). Several of these processes may lead to sustained chronic inflammation driving end-organ disease. Importantly, different clinical phenotypes of long COVID may be driven by different mechanisms, and more than one mechanism may contribute to a particular patient’s condition.

## Current state of long COVID management and potential therapeutic approaches

Currently management of long COVID is focused exclusively on symptomatic treatment, and there is no single acceptable approach. While symptomatic management may benefit patients in the short-term, we believe there is an urgent need to identify treatments that target one or more of the aforementioned pathologic mechanisms (12) in order to better understand the biology, alleviate symptoms, reduce morbidity, and return individuals toward their pre-COVID health. In addition, it is important to identify to Identify biomarkers that can help facilitate development and implementation of therapeutic interventions and monitor the effects of an intervention.

The current state of evidence for various interventions is outlined below. We acknowledge that much of this evidence is derived from small and uncontrolled investigations, thus limiting the ability to evaluate changes that might have occurred over time in the absence of an intervention. While we do not endorse any specific strategy, our goal is to summarize the current state of the science as a call for urgent and efficient research to help the millions who continue to suffer from long COVID around the world.

### Prevention of long COVID

As of October 2022, two COVID-19 vaccines using novel mRNA technology have been approved by the U.S. Food and Drug Administration (FDA): BNT162b2 (Pfizer/BioNTech), and mRNA-1273 (Moderna) and two have been authorized for emergency use including an adenovirus vector vaccine (Janssen), and a protein subunit vaccine (Novavax) (13). These vaccines are safe and effective at protecting people from severe disease, including hospitalizations and deaths (14–16).

There is mounting evidence suggesting a protective effect of vaccination on the incidence of long COVID. Using the TriNetX Research Network platform including 1,578,719 individuals in the USA with confirmed COVID-19, researchers showed that COVID-19 vaccination prevented the occurrence of PASC (17). At 90 days following COVID-19 diagnosis, the incidence of long COVID was lower in the vaccinated cohort than in a propensity score-matched unvaccinated cohort with relative risks of hypertension 0.33 (95% confidence interval [CI], 0.26-0.42), diabetes 0.28 (95% CI, 0.20-0.38), heart disease 0.35 (95% CI, 0.29-0.44), and death 0.21 (95% CI, 0.16-0.27). However, it is clear that SARS-CoV-2 vaccination does not completely eliminate the incidence of long COVID, as a significant proportion of breakthrough infections remain associated with this condition (18).

Another major question is whether treatment during the acute phase of infection with COVID-19 reduces the risk of PASC. Data not yet peer-reviewed from the Veterans Affairs database shows that nirmatrelvir-ritonavir use during the acute phase is associated with a 26% reduction in the risk of developing PASC (19), suggesting that early antiviral treatment might be of benefit. Notably, the data on remdesivir have been less consistent (20, 21). While the focus of this article is on the management of established long COVID, determining whether treatment during the acute phase of infection impacts the risk of developing long COVID will be important in ongoing efforts

### Persistence of viral genetic material and/or antigen

The rationale for the use of antivirals for long COVID treatment is based on evidence suggesting that SARS-CoV-2 may reside in select anatomical reservoirs beyond the initial acute phase, and as such contribute to ongoing inflammation and organ injury. Several studies identified persistence of SARS-CoV-2 genetic material during the post-acute phase. One study described the persistence of SARS-CoV-2 viral RNA shedding for up to 150 days in the upper respiratory tract despite antiviral therapies, with amino acid changes predominantly in the spike gene and the receptor-binding domain leading to viral evolution (22). Another study of 29 patients with long COVID who reported fatigue, muscle pain, dyspnea, inappropriate tachycardia, and low-grade fever found that 13 (45%) had positive plasma RT-PCR results and 51% were positive in at least one RT-PCR sample (plasma, urine, or stool) (23). Another showed that SARS-CoV-2 can be detected in the gut and in the stool for 126 days (24, 25). Other cohorts have identified SARS-CoV-2 proteins during the post-acute phase. For example, a study of individuals with long COVID neuropsychiatric symptoms found both the S1 component of spike and nucleocapsid in neuronal- and astrocytic-derived exosomes (26). In two different cohorts of long COVID patients, SARS-CoV-2 antigenemia was detected in approximately 65% up to 12 months after diagnosis (27, 28). SARS-CoV-2 persistence has also been demonstrated by the presence of genomic RNA, subgenomic RNA, and viral proteins in specific anatomical sites (29, 30) supporting the presence of viral reservoirs in extrapulmonary compartments (24, 30, 31). For example, in one study of autopsy specimens, investigators detected SARS-CoV-2 RNA in multiple anatomical sites including the brain, muscle, gut, and lungs in persons initially diagnosed up to 230 days prior to death (30, 32). These sites were associated with increased inflammatory changes and cytopathological effects. Prolonged gastrointestinal viral shedding for up to seven months, in immunocompetent hosts, and the persistence of SARS-CoV-2 proteins in small and large intestinal mucosal cells correlated with long COVID (24, 25). Although ongoing active viral replication has yet to be confirmed, the growing evidence of persistent antigen and genetic material for months after infection identified in multiple cohorts using multiple mechanisms (27, 28), suggests that the framework that SARS-CoV-2 is a time-limited infection might not be entirely correct.

Although the biological importance of these observations is not yet clear, it remains possible that viral persistence may be an important factor in at least a subset of long COVID cases. An important related question is whether lingering virus directly drives the illness in people experiencing long COVID, or if it induces a dysregulated immune system characterized by heightened release of proinflammatory cytokines that lead to chronic low-grade inflammation and multiorgan symptomatology. This latter mechanism resembles our current understanding of the chronic heightened inflammation seen with chronic HIV infection, which drives several HIV-associated comorbidities (33). For example, people with HIV, even those on suppressive antiretroviral medications, experience ongoing immune activation driven in part by viral persistence in immune privileged sites such as the lymph node (34–36). Over years to decades, this chronic state of immune activation is associated with the acceleration of atherosclerosis, a higher incidence of cardiovascular and pulmonary events, and mild neurocognitive impairment (37, 38). These immunologic complications remain a challenge even in the era of widely available antiretroviral therapy. Similarly, cytomegalovirus infection has broad immunologic effects, including the promotion of immunosenescence and induction of a chronic proinflammatory state (39), and has been associated with cardiovascular disease (40). Other viruses previously thought to be limited to acute illness, including Ebola virus, have been shown to persist in immune privileged sites and cause chronic infection and inflammation (41), providing a framework for persistence that could be evaluated in SARS-CoV-2 infection

While data on the use of antivirals in established long COVID are limited, they support the observation that viral persistence may contribute to some cases of long COVID. For example, there are anecdotal reports of patients whose long COVID symptoms improved following nirmatrelvir-ritonavir administration (42, 43), highlighting the need to study this antiviral agent in controlled and well-designed clinical trials. Two such studies are now underway, evaluating a 15-day course of nirmatrelvir-ritonavir versus placebo in reducing symptom severity in participants with long COVID (44, 45). We believe that studies of antivirals are warranted, and that the sum of the biomarker data, data from treatment during the acute period, and case reports warrant further study of this drug class. This might include currently available antivirals, such as molnupiravir or remdesivir (46, 47), but other agents that have recently been approved in other settings such as ensitrelvir (48) or are under development, e.g. GS441525 (49), could also be considered. Ultimately, the route of administration, side effect profile, and drug-drug interactions will decide which antiviral, if efficacious, is optimal. Monoclonal antibodies may also be worthy of study; however no such agents are currently authorized for treatment of COVID-19 in the United States, due to decreased efficacy *in vitro* against emerging circulating variants (50).

### Therapeutic vaccination for established long COVID

In addition to preventing long COVID, evidence suggests some individuals with established long COVID may benefit from SARS-CoV-2 vaccination. In a meta-analysis of 12 studies evaluating the effects of vaccination on pre-existing long COVID symptoms in 32,726 individuals of whom 8,667 had preexisting signs and symptoms of long COVID. Most of the studies reported improvement in symptoms after one dose, although some reported no benefit or even worsening symptoms (51). Similar observations were noted in other cohorts (52, 53).

In a UK cohort survey of 28,356 participants who previously tested positive for SARS-CoV-2 infection and then received at least one dose of a COVID-19 vaccine, 6629 (23.4%) participants reported long COVID symptoms (presence of symptoms at least 12 weeks after infection) of any severity during follow-up; the first vaccine dose was associated with an initial 12.8% decrease (95% CI −18.6% to −6.6%, P<0.001) in the odds of long COVID, with subsequent data compatible with both increases and decreases in the trajectory (0.3% per week, 95% CI −0.6% to 1.2% per week, P=0.51). A second dose was associated with an initial 8.8% decrease (95% CI −14.1% to −3.1%, P=0.003) in the odds of long COVID, with a subsequent decrease by 0.8% per week (−1.2% to −0.4% per week, P<0.001). While this evidence suggests sustained improvement, the median duration was only 67 days following the second dose suggesting longer follow-up (54).

Postulated hypotheses of the efficacy of the COVID vaccine in established long COVID include the potential correction of dysregulated immune or inflammatory responses or the possible elimination of persisting viruses or viral remnants of SARS-CoV-2, as outlined above (55). Prospective and adequately powered randomized trials are necessary to clarify whether therapeutic immunization will benefit patients with long COVID, and if so, through what mechanism.

### Dysregulation of immune system

Acute SARS-CoV-2 infection is characterized by substantial immune dysregulation, particularly among severe cases, and anti-inflammatory therapy is of benefit in individuals who meet criteria for severe disease (56, 57). Multiple studies have demonstrated that SARS-CoV-2 can result in immunologic perturbations that extend beyond the acute phase of infection (58). Early studies of convalescent plasma donors showed persistence of immune activation in people with prior COVID-19 compared with historical donors (59), and early studies of long COVID showed that elevations in certain biomarkers (e.g., IL-6, TNF-α) during early COVID-19 recovery (1-2 months) were associated with the presence of long COVID symptoms at 4 months (60, 61). Additional studies have shown that individuals with long COVID had highly activated innate immune cells, lack naive T and B cells, and showed elevated expression of type-I IFN (IFN-β) and type-III IFN (IFN-λ1) that remained persistently high at 8 months after infection. A classifier based on cytokine profile that measures IFN-β, PTX3, IFN-γ, IFN-λ2/3 and IL-6 predicted long COVID with 78.5–81.6% accuracy (62). Elevations in levels of IL-6, TNF-alpha, and IL-1B have been consistently reported across multiple studies (63–65), suggesting that these might serve as biomarkers for ongoing disease activity in people with long COVID and that the study of agents that could reduce the levels of these cytokines is warranted.

Data in murine models demonstrated that mice, given coronavirus nasally to mimic a mild infection, develop inflammation in the brain, as well as loss of myelin (66). This model could represent a model of immune dysregulation due to SARS-CoV-2 infection and may allow testing of behavioral and pharmacologic interventions. Studies evaluating anti-inflammatory and immunomodulatory drugs to treat acute SARS-Cov-2 infection have shown benefit, however data is limited for in long COVID (67).

The glucocorticoid receptor acts as a transcription regulatory factor and represses the expression of inflammatory cytokines, chemokines, and prostaglandins, suppresses the antigen-stimulated inflammation mediated by macrophages, dendritic cells, and epithelial cells, and impairs cytotoxic immune responses by downregulating interferon-γ production and inhibiting the development of type-1 helper T cells, CD8+ T cells, and natural killer cells. Thus, glucocorticoids regulate the immune balance between antigen response and inflammation in steady-state and stress conditions (68). The RECOVERY Collaborative Group demonstrated that in patients hospitalized with COVID-19, the use of dexamethasone resulted in lower 28-day mortality among those who were receiving either invasive mechanical ventilation or oxygen alone (69). In a case-control observational study, patients who received oral dexamethasone for hospitalized COVID-19 were also less likely to experience persistent symptoms at 8-month follow-up (70). In a small subset of patients with persistent interstitial lung disease post SARS-CoV-2 infection, treatment with prednisolone was associated with improvement of symptoms, radiological abnormalities, and measures on lung spirometry (71). In a study of 24 patients with abnormal chest CT scans as well as resting hypoxia or exertional desaturation, treatment with deflazacort was associated with decline in breathlessness, tachypnea and hypoxia at rest in patients treated in this uncontrolled case-series (72). Decreased levels of cortisol and cortisone have been observed to be associated with PASC (73). Further data also suggest decreased levels of cortisol among individuals with long COVID (74), but this finding remains to be replicated in other cohorts. Long term use of glucocorticoids results in adverse effects on muscle and bone, increase the risk of osteoporosis and sarcopenia, two severe complications of long COVID (75). These therapies should be investigated in randomized controlled studies, especially given the complicated risk-benefit calculation with long-term use.

There are many other anti-inflammatory interventions that will likely be pursued in future monotherapy and combinatory studies, and these anti-inflammatory agents could target general inflammatory pathways or more specific pathways thought to be important in acute SARS-CoV-2 infection, such as JAK inhibitors, IL-6- or TNF- blockers (57, 76). It is important to note that while targeting the IL-6 pathway has shown clinical benefit in acute COVID-19, and this pathway remains aberrant in long COVID, evidence in favor of biological immunomodulatory therapies such as tocilizumab is limited to a case report (77). As for all immunomodulatory agents, the ease of administration and toxicity profile should be carefully considered in the design of future clinical trials. A full review of the scope of immune system dysregulation and all possible targeting immunomodulatory therapies is beyond the scope of this review.

We believe that such studies testing anti-inflammatory and immunomodulatory therapies are justified based on the current understanding of long COVID pathogenesis. In the meantime, already-available agents with potential anti-inflammatory properties are being repurposed for long COVID. In open-label single-site studies, cohorts treated with low dose naltrexone and low dose aripiprazole have demonstrated clinical benefit among patients with myalgic encephalomyelitis/chronic fatigue syndrome (ME/CFS) (77–79); these medications have subsequently been used off-label for the treatment of long COVID. These agents may represent potential therapies for long COVID due to their anti-inflammatory and immunomodulatory effects, along with a possible impact on amyloid deposition and thrombosis. They are relatively safe, orally active, and of low cost, and therefore, good candidates to be tested as part of therapeutic trials for long COVID.

Low-dose Naltrexone (LDN) is commonly used off-label for people with ME/CFS, a condition characterized by debilitating fatigue and exertional intolerance that has significant clinical overlap with long COVID (78). From a mechanistic perspective, one model of LDN’s efficacy is through suppressive effects on microglia cells of the central nervous system and attenuation of proinflammatory cytokines (80). Microglia are resident macrophages in the brain and spinal cord and normally exist in a resting state but once activated produce proinflammatory factors that interact with neurons to cause hyperalgesia and “sickness response symptoms” such as fatigue, malaise, hypoactivity, sleep changes, etc., which have been demonstrated in animal models (80, 81); there is intense interest in the role that these and other macrophage-derived cells might play in long COVID, particularly in neurocognitive symptoms (82). Additionally, data suggest that the benefits of LDN treatment in ME/CFS are mediated by its regulatory role involving the Transient Receptor Potential Channel Melastatin 3 (83). Other known effects of naltrexone are on Toll-like receptor 4 with downstream effects of re-establishing natural killer cell activation and reducing IL-6, TNF-α and interferon-β levels, pathways that have also been implicated in long COVID (84, 85). Another related mechanistic pathway hypothesized is the possibility of LDN to potentially prevent and treat the immunothrombosis of SARS-CoV-2 and studies are underway to address this question in acute COVID-19 (86). A recently published report evaluated the use of LDN in 38 patients with long COVID, with no control group. In the 36 patients that completed two months of treatment, six of seven evaluated parameters improved over time, with the largest improvements in reduction in pain (79). However, since long COVID symptoms often improve over time, this single-arm study design is insufficient to recommend LDN without further data. A larger study of LDN in 160 participants in a randomized parallel group double-blinded placebo-controlled trial is underway (87).

Aripiprazole is a second-generation antipsychotic agent that was FDA approved in 2002 for use in adult patients with schizophrenia. It has a high affinity for dopamine D2 and D3 receptors as well as for serotonin receptors (88, 89). Besides antipsychotic and antidepressant activities, other pleiotropic properties have been attributed to aripiprazole such as anti-inflammatory actions by decreasing inflammatory and promoting anti-inflammatory cytokines, immunomodulatory effects by a reduction in microglial cell activation and modulation of genes that regulate the immune system, and possible effects on genes implicated in Alzheimer’s disease (90–95). In a retrospective study of 101 patients with ME/CFS, 75 (74%) patients taking low-dose aripiprazole experienced an improvement in one or more symptom categories: fatigue, brain fog, unrefreshing sleep, and frequency of post-exertional malaise (PEM) episodes, or “crashes” (77). These symptoms are frequently reported in patients with long COVID (8, 96, 97), and so further study to assess safety, tolerability, and efficacy in individuals experiencing long COVID are warranted.

Non-pharmacologic approaches are also being pursued for long COVID-associated inflammation. Acupuncture is known to reduce inflammation related to disease and pain, reducing both inflammation and pro-inflammatory cytokines in severe human illnesses such as cancer, multiple sclerosis, and dementia, as well as in allergic respiratory conditions like allergic rhinitis (98). In a preclinical study on the acute phase of burns in rats, acupuncture modulated pain and the inflammatory/proinflammatory cytokine response *via* the downregulation TLR4 signaling pathway (99, 100). Acupuncture may reduce inflammation and provide immune protection *via* heme catabolism and upregulation of heme oxygenase-1 (HO-1) gene expression (101, 102). The HO-1-mediated heme breakdown products (biliverdin, bilirubin and carbon monoxide) exhibit both anti-oxidative and anti-inflammatory effects (103). Acupuncture has been recommended as part of a multidisciplinary team approach for long COVID clinics in some countries, such as the UK, but not in the US (98, 99). RCTs should be conducted prior to routinely recommending this intervention.

### Microthrombi and hypercoagulability

Some investigators have reported the formation of microthrombi, with an increase in α (2)-antiplasmin (α2AP), various fibrinogen chains, as well as Serum Amyloid A, that were trapped in the microcirculation (104), among people experiencing long COVID. Blockage of the microcirculation leads to decreased blood flow, oxygenation, and impairs nutrition delivery and removal of cellular waste (104). In a study of 845 South African people with long COVID, 70 were identified as having laboratory markers consistent with microthrombi (105). A subset of patients (n=24) were treated with one month of dual antiplatelet therapy (clopidogrel 75mg/aspirin 75mg) once a day, as well as a direct oral anticoagulant (apixiban 5 mg) twice a day. The participants in this small case series (not yet peer-reviewed) reported relief of symptoms, primarily fatigue, and biomarker measurements improved including fibrin amyloid microclots and platelet pathology scores (106). These biomarkers need further clinical validation, and given significant potential for harm, anticoagulation or antiplatelet therapies need to be studied in a RCT. The MICHELLE trial (an open-label, multicenter, randomized, controlled trial) showed the use of rivaroxaban versus no anticoagulation for post-discharge thromboprophylaxis after hospitalization for COVID-19, the thromboprophylaxis with rivaroxaban for 35 days an improvement in clinical outcomes (107). If pursued, an ideal clinical trial would restrict this therapy to individuals who have biomarkers consistent with platelet and/or clotting dysregulation and strict medical oversight would be necessary during treatment to mitigate adverse effects, particularly bleeding and gastric inflammation (108).

### Dysbiosis

Changes in the bacterial, fungal, and viral gut microbiome have been reported as a consequence of SARS-CoV-19 infection (109). Patients with prolonged symptoms have demonstrated alterations in the composition of the microbiome with higher levels of *Ruminococcus* and *Bacteroides* and lower levels of *Faecalibacterium* (110). The abundance of genera such as Prevotella and Veillonella are associated with increased inflammation (111). Recently, in a prospective study comparing plant-based fiber or fermented foods in healthy adults, participants consuming a high fermented foods diet had enhanced microbial diversity and a decrease in selected cytokines, chemokines, and other inflammatory serum proteins including IL-6, IL-10, and IL-12b and other inflammatory factors (112). IL-10 is usually considered to be an anti-inflammatory cytokine. However, in some circumstances, IL-10 might be a pro-inflammatory cytokine (113). These results suggest that fermented foods may be powerful modulators of the human microbiome and immune system axis and may provide an alternative to treating post-SARS-CoV-19 associated symptoms. If rigorously studied and demonstrated to be of benefit, dietary interventions could potential be an innovative management approach given the relative ease of implementation. In contrast to the pharmaceutical trial, the dietary clinical trial’s design objective is to measure the impact on health and disease. Some designs include feeding studies, randomized clinical trials, and observational studies. However, nutrition research presents multiple challenges due to the complexity of food environments and the multiple approaches to data collection and analysis, which can influence by the self-reported data, the human nature of the participants, lifestyle, and habits (114, 115).

### Reactivation of other latent viral infections

The vast majority of adults (>90%) harbor infection with latent EBV, a ubiquitous human herpesvirus associated with several post-viral conditions (116). EBV is associated with autoimmune conditions (116), and recent studies have demonstrated links to multiple sclerosis (MS), with a close relationship between EBV seroconversion and development of multiple sclerosis (117), and molecular mimicry between anti-EBNA1 antibodies and anti-GlialCAM antibodies prevalent in MS (118). EBV reactivations may also contribute to immune dysregulation.

One early study of long COVID made the observation that two-thirds of individuals experiencing long COVID symptoms demonstrated EBV early antigen-diffuse (EA-D) IgG positivity, suggesting reactivation around the time of SARS-CoV-2 infection (119). More recent efforts demonstrated that the presence of EBV DNA during the acute phase of COVID-19 predicted the presence of symptoms 30-60 days later (73). Another recent larger study of primarily outpatients found that a higher proportion of participants who experienced long COVID had evidence of high-level EBNA IgG levels (47% versus 28%; P<0.05); participants with detectable EBV EA-D IgG responses had a 2.12-fold higher likelihood of post-COVID fatigue, and participants with high levels of EBV nuclear antigen (EBNA) IgG levels (>600 U/mL) had a 2.5-fold higher likelihood of long COVID neurocognitive symptoms (120). These findings are consistent with other post-COVID cohorts (119), and suggest that further investigation of the relationship between EBV-related pathology and long COVID is warranted.

EBV reactivation has also been proposed as a driver of ME/CFS (121–124). One single-arm, uncontrolled case series showed that 9/12 (75%) persons with ME/CFS after treatment with valganciclovir experienced symptomatic improvement allowing return to usual activities; this was sustained off treatment (125). Another retrospective study showed that 52% of patients receiving daily valganciclovir experienced >30% improvement in physical and/or cognitive function (126). In uncontrolled studies such as these, we cannot determine what percent would have improved without treatment. A follow-up randomized study showed improvement in fatigue and cognitive function; those in the intervention group were 7.4 times more likely to respond in this small study (126).

As a result of these observations, there is now growing interest in anti-EBV therapies to modulate disease morbidity and improve symptoms in long COVID. Therapies currently being considered include antivirals like valganciclovir, a guanosine analog with potent anti-human herpesvirus activity, including *in vitro* and *in vivo* activity against the lytic phase of EBV that can reduce mucosal shedding of EBV during treatment (127). Other therapies under consideration are considerably more complex, including anti-B cell therapeutics (e.g., rituxmimab) or CAR-T cells. While few clinical trials addressing these potential targets are active as of the time of this writing (128, 129), Bateman Horne Center is running a Phase 2a, open-label trial of Virios Therapeutics’ IMC-2, a combination of valacyclovir and the nonsteroidal anti-inflammatory drug celecoxib, for long COVID symptoms potentially driven by herpesvirus reactivation (130).

Interestingly, despite the associations between latent CMV infection and chronic immune activation, one recent study found that CMV-seropositive individuals had a lower odds of certain types of PASC symptoms, particularly neurocognitive symptoms (131). This pattern was the opposite of what was seen with evidence of recent EBV reactivation. The reason for this observation is unclear, but the authors proposed that it could be related to different immunologic compartmentalization of each of the latent viruses. For example, they suggested that CMV-associated immunoregulatory pathways could decrease inflammation and modulate other potential pathophysiologic mechanisms (e.g., autoantibody formation) (132). In addition, they speculated that CMV seropositivity could be associated with heightened adaptive immune responses as it is with regard to influenza vaccination (133). More work will be needed to determine what role, if any, CMV serostatus plays in the pathogenesis of PASC.

### Fibrosis

Residual pulmonary disease is a well described complication of viral pneumonitis which can lead to fibrosis. Myall and colleagues reported that 39% of 837 patients still had respiratory symptoms 4 weeks post discharge following SARS-CoV-2 infection (71). Dyspnea can be secondary to multiple etiologies including alveolar damage with resultant airway fibrosis, pulmonary thrombosis and bronchiolitis (134). Nintendanib and pirfenidone are approved for the treatment of idiopathic pulmonary fibrosis and have both been proposed as treatment for fibrotic lung disease secondary to COVID-19 pneumonia (135). A phase 2 placebo-controlled trial of pirfenidone for post COVID-19 pulmonary fibrosis is ongoing (136). The PINCER trial compares nintendanib with pirfenindone in persons with post COVID-19 pulmonary fibrosis (137). Given that bronchiolitis with air trapping is commonly seen in long COVID (134, 138), clinical trials of an anti-fibrotic targeted to treat constrictive bronchiolitis to prevent fibrosis development should be considered in persons with persistent dyspnea and cough without radiographic evidence of fibrosis. Enrollment of 168 participants in a placebo-controlled trial evaluating deupirfenidone (LYT-100) for treatment of post acute COVID-19 respiratory complications was completed July 2022 with results expected early 2023 (139).

### Sleep disturbances and post exertional malaise/fatigue

Long COVID symptoms may include anxiety, depression, brain fog, and sleep disturbances (140, 141). While sleep disturbances are common and more recently have been attributed to societal restrictions during the COVID-19 pandemic, a systematic review and meta-analysis revealed that over 50% of people who had been infected with SARS-CoV-2 suffered with sleep disturbances (142). One small study demonstrated significant sleep impairment four months after having COVID-19 with an increased prevalence among those with obstructive sleep apnea (143). Chronotherapy is an emerging therapeutic in pulmonary and sleep medicine as we understand how the disruption of circadian rhythm affects cells of virally infected organs such as the lung and the immune system (144). Therapies directed at improving sleep and recalibrating circadian rhythm by light therapy, cognitive behavioral therapy for insomnia and melatonin are currently being studied for the sleep disturbances due to the pandemic but have not been initiated yet for long COVID (145).

Post exertional malaise (PEM), also called “crash,” has been clinically defined as an increase in fatigue, pain, cognitive dysfunction, and flu-like illness after physical exertion, mental activities, or stressful events (146, 147). A recent meta-analysis found that PEM is 10.4 times more likely to be associated with ME/CFS (148). Therefore, PEM has been considered the hallmark for diagnosing ME/CFS (146–148). Some patients with long COVID experience similar clinical PEM symptoms. In a pre-print manuscript, investigators described a cohort of 105 patients from the Stanford long COVID clinic with symptoms greater than six months; they found that fatigue, PEM, and brain fog were the predominant and severe symptoms of which 43% were diagnosed with ME/CFS (149). PEM can be mitigated by energy conservation activities known as ‘pacing’ (resting, decreasing stress or overstimulation) (150). A supervised activity program may involve brief walking (~5 minutes), stretching/limb and spine active range of motion, participation in personal or instrumental activities of living, or reading for 5-10 minutes. The duration of these activities is then slowly increased. This method may allow careful introduction of aerobic exercise to prevent deconditioning (cycling, tai chi, yoga or aquatic exercises). For all patients, the goal of this individualized activity program is to regain functional independence, resume participation in life roles, and restore of quality of life (151).

### Mitochondrial health

At least one study has identified changes in mitochondrial health to be associated with long COVID (26). AXA1125 is a mixture of six amino acids that can increase fatty acid oxidation, ATP production, ketogenesis, and mitochondrial bioenergetics leading to improved muscle function. Promising Phase 2a results were announced by the company Axcella Health in the third quarter of 2022 for its trial of AXA1125 which targeted fatigue predominant long COVID (152). This small randomized, double-blind, placebo-controlled trial showed significant improvements in fatigue, as measured by the Chalder Fatigue Questionnaire eleven-item scale and in the six-minute walk test endpoint (153). However, the study did not show a significant improvement of mitochondrial function by magnetic resonance spectroscopy. A phase III study is planned and under development. A recently conducted RCT of co-enzyme Q for long COVID did not show an effect (154).

## Discussion

Long COVID is now recognized as a major clinical challenge limiting the return to baseline health of a substantial number of people following SARS-CoV-2 infection. While clinical trials were rapidly implemented and conducted to determine the optimal management of acute COVID-19, therapeutic studies for Long COVID have lagged behind. This has occurred for a variety of reasons, including delays in the medical community’s recognition of the clinical significance of the condition as well as the complex and likely multifactorial nature of its pathophysiology, which is still being elucidated. We are grateful that the NIH has made a substantial investment in long COVID and are hopeful that other funders, including industry and philanthropic organizations, will follow suit. It is likely that multiple concurrent and coordinated efforts will be needed to accelerate progress in further defining and understanding this condition.

Although major efforts are now underway to understand the biology of long COVID, the current evidence is limited. At the same time, patients, their clinicians, and their advocates are eager to identify treatments that can provide symptomatic relief and perhaps even reverse the underlying pathophysiology of this condition. The desire to treat and alleviate suffering versus the need to rigorously test such interventions represents a fundamental tension for the field. While we can learn from treatment anecdotes, the case reports, series, and small, uncontrolled studies conducted and reported to date must be interpreted with great caution, as their design makes it impossible to discern whether an intervention was responsible for the clinical changes observed in patients or participants. This is further complicated by the fact that the natural history of long COVID is characterized by within-individual variability in symptoms (which can wax and wane) and by the fact that individuals experiencing long COVID may improve over time even in the absence of an intervention.

We recognize that many individuals experiencing this condition are eager to return to their baseline state of health. Such individuals may choose to work with their care providers to devise a treatment plan that makes use of unapproved or off-label therapies after careful consideration of and counseling regarding the potential risks and benefits. However, it is our position that it is not currently possible to endorse any particular treatment approach aside from seeking clinical care where indicated. Rather, it is our goal to emphasize the urgency for the implementation of well-designed clinical trials that would serve two purposes: to further define the pathophysiology of long COVID and to determine the efficacy and safety of candidate treatments.

There are many important considerations for long COVID clinical trials. Beyond the intervention to be evaluated, those designing such studies must consider whether the study should be focused on one or more long COVID phenotype or enroll more broadly, what phase after SARS-CoV-2 infection is the target for intervention (acute, early post-acute, post-acute), the optimal design and if relevant, randomization scheme, and the optimal outcomes, which in most cases are likely to involve a combination of patient-reported outcomes and biological measurements. While our group has diverse opinions on which types of studies are optimal and which specific agents should be prioritized, we believe that a variety of designs could have a role. For example, a small, proof-of-concept study of an intensive therapy might focus on biological measurements in a few dozen individuals to further define the role of a mechanistic pathway, while a large, randomized trial might seek to determine whether a scalable intervention has an effect on symptom outcomes in a cohort of hundreds or thousands of volunteers. Both approaches, and all those in between, potentially have merit. We believe there is great urgency for these studies to be performed and are encouraged by the recent or planned launch in the near future of several clinical trials, including those through the RECOVER initiative. However, given the complexity of the condition, we believe that ultimately there will need to be ongoing, concerted efforts between people experiencing long COVID, regulators, academic and industry researchers, and funders (including funders outside RECOVER) to get a greater variety of studies into the clinic in order to obtain the answers that patients and clinicians are seeking in a reasonable timeline.

While we sought to be comprehensive, this report has several notable limitations. First, there are numerous mechanisms of long COVID and it is beyond the scope of this review to exhaustively review all hypothesized mechanisms in this article. Second, it is possible that many of the mechanisms we discussed are inter-related and thus a single intervention may in the end address several mechanistic pathways. Third, we do not discuss pathophysiology or interventions in certain subpopulations of interest, including children and pregnant people. While there may be overlap with presentations of long COVID in adults, appropriate management of long COVID in children, including MIS-C, requires consideration of pathophysiology, biology, pharmacology, neurocognitive and behavioral development unique to children (155), which is beyond the scope of this article. Given the paucity of studies evaluating interventions to help children and adolescents with long COVID, clinical trials that leverage approved interventions that pose the sufficient prospect of benefit to justify any associated risks such as antivirals, vaccines, and monoclonal antibodies as well as physical rehabilitation and mental health regimens should be prioritized. Similarly, unique pathophysiology may contribute to long COVID in pregnant people and there are unique risk and benefit considerations during the intra- and post-partum periods. Fourth, we do not consider the costs or complexity of the discussed interventions, although in general we focused on treatments that we believe to be generally practical. Fifth, we do not provide a direct discussion of how these interventions should be prioritized in relation to one another, as that is beyond the scope of our taskforce’s charge. Finally, this is a fast-moving field with new observations and data released on an almost daily basis; such data should be considered in addition to the content of this article for those pursuing clinical trials.

Involving patients, caregivers, and community representatives in intervention research and decision making is crucial. In the instance of Long COVID we are also faced with a novel condition where treatments are still being determined. Patients and caregivers offer the unique and important perspective of living daily with the challenges of Long COVID and can therefore provide key insight into essential topics such as the most common and bothersome symptoms, or which treatment outcomes could offer the best opportunity for improved quality of life. By including patients, caregivers, and community representatives in as many aspects of the process as possible we are creating a collaborative environment which increases the likelihood of better outcomes, strengthens patient trust and acceptance, and broadens our overall understanding of the condition and best intervention approaches.

Enrolling a diverse population of patients can ensure adequate representation and improve access to care for those individuals who are disproportionately impacted by COVID-19 and may be faced with limited availability of vaccines and treatments. This ensures a broader scope of the issue and can even help to uncover hidden disparities or barriers to care which can further guide the most impactful and needed interventions for patients. Engaging diverse populations in research and interventions also expands access to education that can inform both the patients and providers, creating mutually beneficial learning opportunities. Inclusivity also builds trust within the patient and caregiver community, while fostering a sense of belonging that can deepen patient satisfaction and lead to better health outcomes.

## Conclusion

As the COVID-19 pandemic continues, substantial numbers of individuals will continue to experience long COVID. Given the vast number of individuals infected by this virus, the magnitude of this health crisis is easily appreciated. Consequently, it is critical that research extend beyond the many important and informative epidemiologic studies describing the extent of the problem to include trials of interventions to prevent, treat or ameliorate the varied symptoms and diverse manifestations associated with long COVID. Although the pathophysiologic basis of long COVID remains incompletely understood and the clinical spectrum of the condition is quite diverse, evidenced-based theories have been proposed, providing potential plausible targets for intervention. This paper describes the early work assessing several potential therapies aimed at preventing and treating long COVID and its various manifestations. It is unknown which, if any of these potential therapies will have an impact alone or in combination with other interventions on the course of long COVID. Moreover, different clinical phenotypes of long COVID may be driven by different mechanisms, and effective therapies may vary from one patient to the next. However, it is established that such therapies are needed and more work in this area is necessary. The current data are limited by the fact that most studies to date are small and uncontrolled, and therefore limited in their ability to evaluate changes over time that might have occurred without intervention. This paper highlights the critical need for timely and efficient research strategies to help the millions suffering from long COVID around the world.

## Author contributions

Long COVID is now recognized as a major contributor to post-COVID morbidity and delayed return to health. This article represents the current state of understanding regarding long COVID pathogenesis and therapeutics, with a view toward interventions that the authors believe warrant further evaluation in well-designed clinical trials. It provides an overview of the current state of evidence and proposes a pathway forward for efforts to address this new set of conditions which are anticipated to continue to impact the lives of many individuals recovering from SARS-CoV-2 infection and are already putting a major strain on healthcare systems. All authors contributed to the article and approved the submitted version.
